# Supplementary material for: The Affective Ising Model: A computational account of human affect dynamics
Source: PLoS Comput Biol. 2020 May 15;16(5):e1007860. doi: 10.1371/journal.pcbi.1007860 (PMC7255618; doi:10.1371/journal.pcbi.1007860)
Supplement: S1 Appendix — In this appendix, the numerical procedure that was used to fit the AIM is explained in detail, which is similar to a procedure used by [60]. (PDF) [file pcbi.1007860.s001.pdf]

Appendix S1: Fitting the AIM

Computing the likelihood

The emotion items are aggregated at the momentary level by averaging over the positive and negative emotion items respectively. This results in a bivariate time series of PA and NA measurements (varying between 0 and 1).

In order to compute the min-log-likelihood, the time series is transformed into a list of consecutive measurements $\{(\mathbf{y}(t_i), \mathbf{y}(t_{i+1}))\}^{i=1,\ldots,n}$. In this notation, $\mathbf{y}(t_i)$ corresponds to a two-dimensional vector consisting of the PA and NA scores at the measurement occasion labeled by t_i. n denotes the number measurement occasions. The data is formatted in this way because the AIM is Markovian, meaning that only the previous measurement is required to compute the likelihood of a specific observation. The min-log-likelihood is the sum of the negative logarithms of all these individual likelihoods.

Given a set of model parameters $\hat{\mathbf{\Theta}}$, the likelihood of an observation $\mathbf{y}(t_i)$ corresponds to the conditional probability of making that observation given $\hat{\mathbf{\Theta}}$ and the previous observation $\mathbf{y}(t_{i-1})$. For the AIM, there is no analytic expression for these conditional probabilities. Therefore, a numerical method has to be used. There exist several numerical techniques to compute such conditional probabilities but for this paper a similar technique was used as the one described in [60]. The reason for this is that their algorithm can be made to run on a Graphics Processing Unit (GPU) which drastically speeds up the computations. The idea is to discretize both time and the affect space. The two-dimensional affect space is divided into a grid in which all neighboring points are equidistant. Time is also no longer continuous but evolves in discrete steps Δt. A differential operator $\mathcal{H}$ is constructed based on the dynamical equations Eq (3) which describes how a probability distribution diffuses on the affect grid during a discrete time step Δt, starting from a specific observation. In other words, starting from a Dirac-delta peak at the observation $\mathbf{y}(t_i)$, the approximate conditional probability distribution is obtained for time $t_i + \Delta t$ by operating on it with $\mathcal{H}$. Upon

repeating this, the probability distribution is obtained for time $t_i + 2\Delta t$. To obtain the probability distribution at time t_{i+1}, this procedure is repeated $N = (t_{i+1} - t_i)/\Delta t$ times. If N is not an integer, the probability density can be obtained by interpolating between the density after $M - 1$ and M steps, where M denotes the integer for which $M - 1 < N < M$.

Note that all measurements $\mathbf{y}(t_i)$ appear as an end point of a pair in the list of consecutive measurements except for $i = 1$. There is no information prior to the first measurement. The likelihood of this observation is therefore computed using the equilibrium distribution.

Bridging nights can be time consuming. For the studies used in this paper, the time interval between the last measurement of a day and the first measurement of the next was at least 12 hours. This is fairly large in comparison to the average of 1.5 hours between two consecutive daytime measurements. To avoid having to simulate a humongous number of time steps during the nights, all measurement pairs connecting the last measurement of a day with the first of the next are also left out of the list. The likelihood of every first measurement of a day is computed using the equilibrium distribution of the model. This is justified in light of the typical autocorrelation which is small enough for the affect system to relax to equilibrium overnight.

Finding the min-log-likelihood estimates

The model estimates are computed using the maximum-likelihood technique. In other words, the fitted parameters corresponds to the maximum-likelihood estimates. These can be found by minimizing the min-log-likelihood. There is no analytic expression for the min-log-likelihood function of the AIM and there is especially no analytic expression for its maximum-likelihood estimates. Because the min-log-likelihood function may have multiple local minima, a global optimization heuristic was used to obtain the estimates of the datasets used in this paper.

The algorithm that was used is the Differential Evolution (DE), which is considered to be a simple and efficient heuristic for global optimization over continuous spaces [58]. It relies on evolutionary concepts, such as mutation, crossover and selection, to obtain an optimal candidate. In this paper, the typical DE parameters that were used are: a

DE population $NP = 50$, a crossover probability $CR = 0.7$ and the *binomial DE/rand/1* crossover strategy. The number of DE iterations typically lied around 1,000.

The DE algorithm is only a heuristic; there is never a guarantee that the global minimum has been found. The optimization procedure was therefore repeated 50 times for each dataset, each time with a different initialization. Out of the 50 runs, the estimate with the smallest min-log-likelihood was retained.

Testing the procedure on simulated data

A recovery study and a coverage study was done to test the fitting procedure. By means of the recovery study it was investigated whether the exact parameters with which data had been simulated could be retrieved. This was investigated for various sample sizes. The coverage study, on the other hand, was used to examine the confidence intervals around the parameters.

Both studies relied on data simulation. Therefore, in first instance, the simulation method is explained. Then, the actual studies and their results are discussed.

Simulating data

To simulate data, parameters are required. In order to cover a (more) relevant part of the parameter space, 300 datasets were randomly selected and the corresponding maximum-likelihood estimates were computed using the fitting procedure discussed above. Although the trustworthiness of the procedure had not yet been verified when obtaining these estimates, they still ensured a more realistic simulation study; at least more realistic than a random selection of parameters. These 300 parameter vectors were subsequently treated as true parameter vectors (denotes as $\boldsymbol{\Theta}$) and they were used to simulate data.

Because the data format is that of a time series, a second ingredient that is required to simulate data is a schedule of measurement moment (i.e., beeps). Again, to ensure relevant schedules, those of the actual datasets were used.

To simulate data, the observed data points in the dataset, except the last one, were used as starting points. Eq (3) in combination with the parameter vector $\boldsymbol{\Theta}$ corresponding to the dataset were used to simulate a data point at the time of the

ensuing beep. An observation at the time of the very first beep was obtained using the equilibrium distribution Eq (1). To avoid burdensome computations, the first measurement of each day was also drawn from the equilibrium distribution instead of running a simulation from the last beep of the previous day.

Manipulating the sample size

For both the recovery study and the coverage study a range of sample sizes were used. The larger the number of data points, the easier it must become to recover the parameters with which the data was simulated. Retrieval must be exact for an infinite amount of data. Similarly for the coverage study, not only should the confidence intervals of the parameters become smaller with an increasing number of data points, but any discrepancy between the actual coverage probabilities and the nominal coverage probabilities should become smaller as well.

To manipulate the sample size without increasing the computational burden of the fitting procedure, instead of sampling only one observation at the time of the ensuing beep, multiple data points are drawn from the simulated conditional probability distribution (which can also be the equilibrium distribution). Thus, instead of sampling only one data point from each simulated distribution, N data points are sampled. The number of data points obtained in this manner is N times the number of beeps in the original dataset. The number of likelihoods that have to be computed during optimization is nonetheless unaffected because the evaluations of all the end points can be done simultaneously. N can be considered as a multiplication factor. For $N = 1$, the simulated dataset has as many observations as the original dataset (typically 70). For $N = 100$, the simulated dataset is a hundred times larges than the original one.

Recovery

The results of the recovery study for the AIM are shown in Fig A. The recovered parameters are depicted as a function of the true parameters that were used to simulate data for $N = 1$ (lighter dots) and $N = 100$ (darker dots). If the recovery is exact, the dots lie on the main diagonal (red line). We can see that the recovery becomes exact as the sample size increases, except for the parameter D. For the parameter D, there is an issue with large values; the larger the value of D, the quicker the model evolves towards

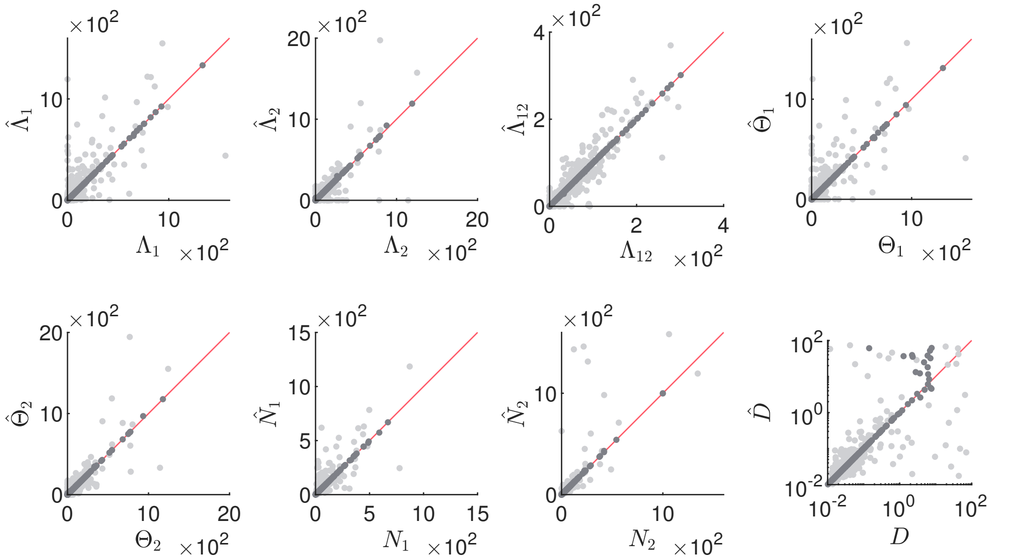

Fig A. Results of the recovery study for the AIM.
The recovered parameters are depicted in function of the true parameters. If the parameter was recovered correctly, the point lies on the main diagonal. Lighter dots correspond to a recovery study with $N = 1$ and darker dots correspond to a study with $N = 100$.

its equilibrium (i.e., almost no autocorrelation). When the D becomes too large, the model will always relax towards its equilibrium distribution before it reaches the next beep. When this happens, the actual value of the D is not important anymore. It only matters that it is sufficiently large.

Coverage

We used a coverage study to examine the frequency with which the true parameter values were contained in the estimated confidence intervals with a predetermined confidence level. If, for instance, the confidence level was set at .95, the confidence intervals should cover the true parameters in 95% of the cases. This is referred to as the *nominal coverage probability*. The *(actual) coverage probability* is defined as the proportion of true parameters that actually fall within of the computed confidence bounds. Ideally, the actual and nominal coverage probabilities are equal.

For the coverage study, data were simulated with $N = 1$ and $N = 10$ (it is not meaningful to make the sample size too large because then the confidence intervals become very narrow). For both sample sizes, the actual coverage probabilities were obtained by means of a parametric bootstrap (with 200 replications). In Fig B the

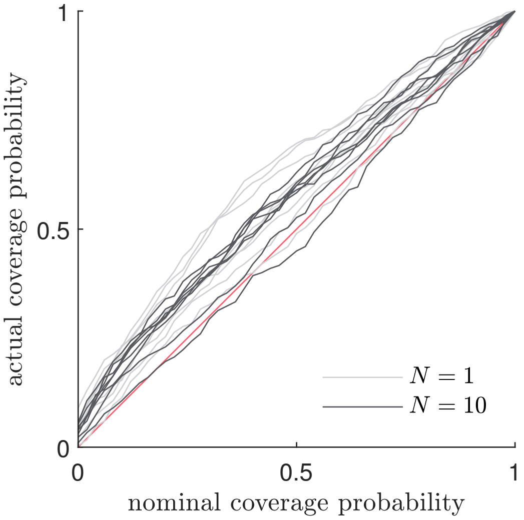

Fig B. Results of the coverage study for the AIM.
The actual coverage probabilities of the eight AIM parameters are depicted in function of the nominal coverage probability for $N = 1$ (light) and $N = 10$ (dark). The main diagonal (red) denotes the expected functional relation between the actual and the expected coverage probabilities.

actual coverage probabilities are set out against the nominal coverage probabilities. The lighter lines correspond to $N = 1$ and the darker lines to $N = 10$. All of these lines are expected to lie on the main diagonal indicated in red. As the sample size increases, the lines shift more towards the main diagonal. In general though, the actual coverage probability is larger than the nominal coverage probability, meaning that the computed confidence intervals are somewhat too broad. This overestimation is small in the upper right corner however (which practically matters the most). Hence, we can conclude that the coverage performance of the AIM is sufficient.

The bounded OU model

To compute the likelihoods of the bounded OU model the same simulation based technique was used as the one that was used for the AIM. As a sanity check, we also did a recovery study for this model. The results turned out to be similar to those of AIM.
