# Supplementary material for: The Affective Ising Model: A computational account of human affect dynamics
Source: PLoS Comput Biol. 2020 May 15;16(5):e1007860. doi: 10.1371/journal.pcbi.1007860 (PMC7255618; doi:10.1371/journal.pcbi.1007860)
Supplement: S2 Appendix — In this appendix, the method for analyzing the three summary statistics, autocorrelation, PA-NA correlation [61] and the root-mean-square of successive differences [4], is described. The results of the analysis are discussed as well. (PDF) [file pcbi.1007860.s002.pdf]

Appendix S2: Reproducing common summary statistics

In this appendix, we discuss how we evaluated to which extent the models can capture the common summary statistics: autocorrelation, PA-NA correlation and RMSSD.

Autocorrelation. The autocorrelation τ was assessed through the Pearson correlation between consecutive measurements. There are separate autocorrelations for the PA and NA dimensions. Measurements that became consecutive because of missing data were not included in the computation of the Pearson correlation, since their time intervals are disproportional to the other time intervals. For the same reason, nights were excluded.

Additionally, we evaluated the importance of the actual time dynamics by comparing the AIM with an equilibrium version of itself. This so-called equilibrium version of the AIM is simply the AIM without any autocorrelation; all data points are sampled from the equilibrium distribution. This corresponds to a model in which the affect system always entirely relaxes before the next measurement occasion. If this is the case, no information about the specific time dynamics can be derived from the data and an equilibrium model should be sufficient to describe the observed pattern. The leave-one-out likelihoods should then be smaller for the equilibrium model.

PA-NA correlation. The PA-NA correlation ρ is simply the Pearson correlation between concurrent PA and NA scores [61].

RMSSD. The mean-square successive difference (MSSD; [4]) is defined as the average of the squared differences between the scores of subsequent measurements. The root of the MSSD is a measure of the average jump between consecutive measurements.

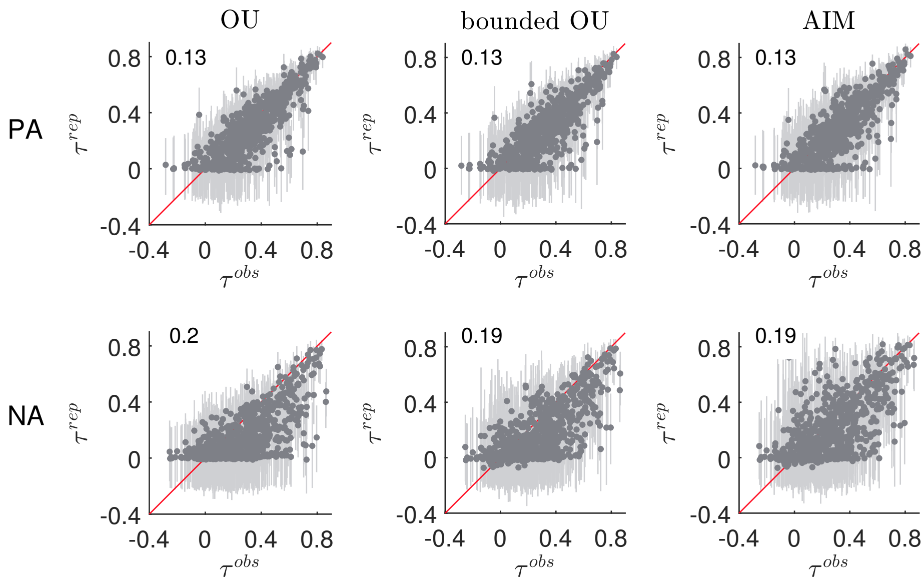

Fig A. Autocorrelation results.
The medians of the replicated autocorrelations τ^{rep} set out against the observed autocorrelations τ^{obs} for the different datasets. The lighter bars correspond to the 90% confidence intervals based on the distribution of τ^{rep}. The first, second and third columns respectively correspond to replications based on the OU model, the bounded OU model and the AIM. The root-mean-square error between the median and observed autocorrelations is depicted in the upper left corner of each panel.

Results

Autocorrelation

The results of the parametric bootstrap of the autocorrelations τ are shown in Fig A. The structure and information content of the figure is the same as for Fig 5 in the paper.

Observed autocorrelations range between -0.25 and 0.84 for the PA dimension, and -0.25 and 0.86 for the NA dimension. The average autocorrelation is equal to 0.32 for the PA dimension and 0.28 for the NA dimension. In general, all three models do comparably well in retrieving the observed autocorrelation. They are able to reproduce the full range of autocorrelation, except for the negative ones. The models can give rise to data patterns with negative autocorrelations, as can be deduced from the lighter bars dropping below zero, but the medians themselves do no really drop below zero. This indicates that it is difficult for the models to produce such datasets. Yet, 5% and 10% of the data patterns exhibit negative autocorrelations in the PA and NA dimensions respectively. It should be noted, however, that the absolute values of these negative

autocorrelations are typically small and could be spurious.

There is little difference between the models for the PA dimension, as can be deduced from the panels in the first row of Fig A. For the NA dimension, the OU model seems to typically underestimate the autocorrelation; the majority of points in the lower right panel of Fig A lie under the diagonal. For the bounded OU model, the points partly shift upwards and even more so for the AIM. The root-mean-square errors between the median replicated autocorrelations and the observed autocorrelations are nevertheless comparable for all three models.

For about half of the datasets the largest autocorrelation in either dimension is smaller than 0.3. When the autocorrelation is small, the models will generally tend to relax to their equilibrium distribution more rapidly. Obviously, at some point an equilibrium model will suffice to describe the observed pattern. According to the leave-one-out, the equilibrium AIM seems to be sufficient for 39% of the datasets when compared to the standard AIM. Similar results hold for both the OU and bounded OU models. In all three cases, the distribution of leave-one-out likelihoods is nevertheless heavily skewed in the direction of the dynamical model, indicating that there are datasets for which a dynamical model clearly performs better. The converse is not true however; whenever the equilibrium model outperforms the dynamical model, their difference is relatively small.

In Fig B and Fig C, a day out of the lives of two different participants is shown. The autocorrelation of the time series in Fig B is large in both the PA and NA dimension, and the AIM nicely picks up on this autocorrelation. For the time series in Fig C, the AIM is not able to reproduce the observed autocorrelation; the model always immediately relaxes after each measurement, there is no autocorrelation at all. Such discrepancies between model fit and reality occur for all three models. In the panel of Fig A they are represented by the dots on the horizontal axis with $\tau^{rep} = 0$.

In conclusion, the data incorporate a broad range of autocorrelations. The three model are capable of reproducing this broad range of autocorrelations and do so comparably well. For a large proportion of the datasets, the observed autocorrelation is small; for 39% of them a model without explicit time dynamics would suffice.

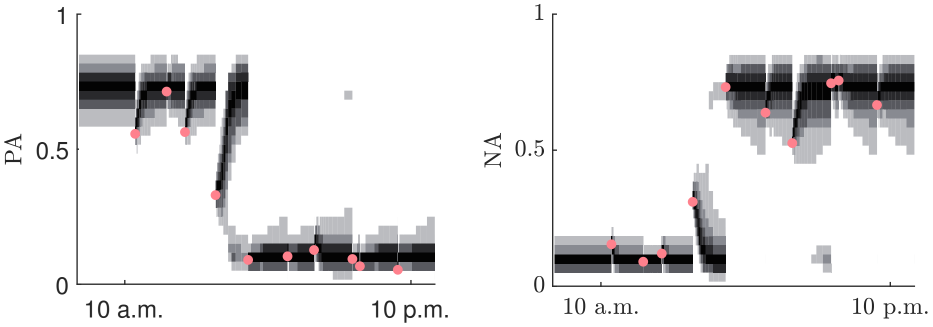

Fig B. A day out of the life of one of the participants.
The left panel shows the PA scores across time and the right panel shows the NA scores. The marginalized affect distributions as predicted by the AIM are depicted as density maps; the darker, the more dense the probability distribution. The observed autocorrelation is 0.75 for both the PA and NA dimensions.

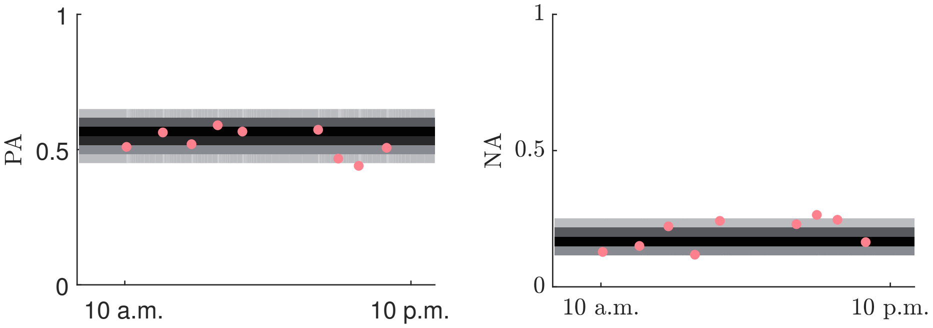

Fig C. Another day out of the life of one of the participants.
The left panel shows the PA scores across time and the right panel shows the NA scores. The marginalized affect distributions as predicted by the AIM are depicted as density maps; the darker, the more dense the probability distribution. The observed autocorrelations are 0.24 and 0.63 for the PA and NA dimensions respectively. The autocorrelations predicted by the AIM are nonetheless close to zero.

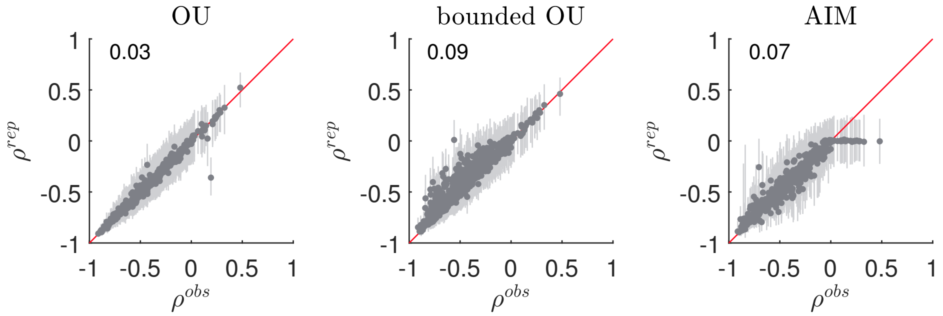

Fig D. PA-NA correlation results.
The medians of the replicated PA-NA correlations ρ^{rep} set out against the observed PA-NA correlations ρ^{obs} for the different datasets. The lighter bars correspond to the 90% confidence intervals based on the distribution of ρ^{rep}. The first, second and third columns respectively correspond to replications based on the OU model, the bounded OU model and the AIM. The root-mean-square error between the median and observed PA-NA correlations are shown in the upper left corner of each panel.

PA-NA correlation

The results of the parametric bootstrap of the PA-NA correlations ρ are shown in Fig D. The structure and information content of the figure is the same as for Fig 5 in the paper.

The PA-NA correlation is a parameter included in (lag-1) vector autoregressive models. This measure should therefore be trivially recovered using such a model. As can be seen in the first panel of Fig D, the transition from discrete to continuous time does not destroy this trivial relation between statistical measure and model parameter; the covariance of the PA and NA scores determines the covariance of the Gaussian distribution that best fits the data. The recovery is almost exact for the OU model. The root-mean-square error between the median model based PA-NA correlations and the observed PA-NA correlations is 0.03.

In Fig D we see that cutting off the Gaussian distributions breaches the trivial relation between observed and simulated PA-NA correlation. The recovery becomes less exact, but the majority of points still lies close to the main diagonal. There are however many points that have shifted above the diagonal, indicating that the bounded OU model sometimes underestimates the observed negative correlation. The root-mean-square error is 0.09.

The spread on the replicated PA-NA correlations is also larger for the AIM than for the OU model. In contrast to the replicated correlations of the bounded OU model, those of the AIM are spread out symmetrically on both sides of the main diagonal. The

dots in the third panel of Fig D however diverge from the main diagonal when the observed correlations become larger than zero. The AIM, having been constrained to only allow inhibitory interactions between the PA and NA dimension, seems to be unable to yield the observed positive PA-NA correlations. In spite of this, the root-mean-square error is only 0.07.

In conclusion, the OU model almost exactly reproduces the observed PA-NA correlations. For the bounded OU model and the AIM, the reproduced values are also close to the true values but less exact. The reproduced values of the bounded OU model are a bit biased though, and the AIM cannot reproduce positive PA-NA correlations. From a theoretical perspective, a positive correlation between the PA and NA dimension is peculiar. Hence, the AIM not being able to produce such correlations could be seen as a benefit.

RMSSD

The results of the parametric bootstrap of the root-mean-square successive differences are shown in Fig E. The structure and information content of the figure is the same as for Fig 5 in the paper.

When it comes to reproducing the root-mean-square successive differences, the three models perform comparably well. The root-mean-square error between median and observed RMSSD is identical for all models and dimensions. No major misfit can be observed.

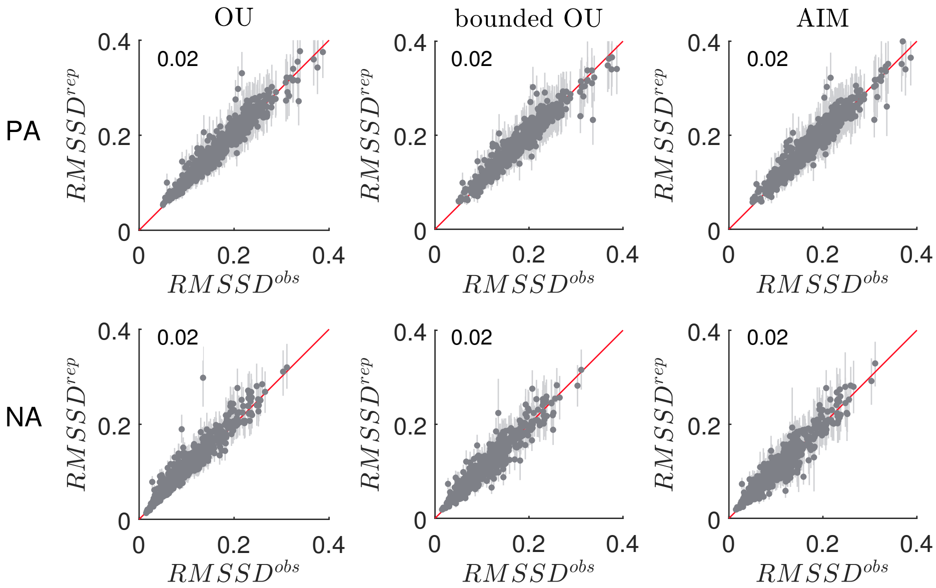

Fig E. RMSSD results.
The medians of the replicated root-mean-square successive differences RMSSD^{rep} set out against the observed root-mean-square successive differences RMSSD^{obs} for the different datasets. The lighter bars correspond to the 90% confidence intervals based on the distribution of $RMSSD^{rep}$. The first, second and third columns respectively correspond to replications based on the OU model, the bounded OU model and the AIM. The root-mean-square error between the median and observed root-mean-square successive differences are shown in the upper left corner of each panel.
