# Supplementary material for: The Affective Ising Model: A computational account of human affect dynamics
Source: PLoS Comput Biol. 2020 May 15;16(5):e1007860. doi: 10.1371/journal.pcbi.1007860 (PMC7255618; doi:10.1371/journal.pcbi.1007860)
Supplement: Attachment — Submitted filename: Responses.pdf [file pcbi.1007860.s003.pdf]

Responses to reviewers

Reviewer #1

This article proposes a nonlinear stochastic model for studying the dynamics of affect in humans, labeled by the authors the Affective Ising Model (AIM). The model is based on neurophysiological and behavioral fundamentals underlying positive and negative affect, and is a model that can account for various types of manifestations of affect such as skewness, multimodality, and non-linear relations of positive and negative affect. As part of their illustration, the authors implement the model to two empirical data sets of affect from experience sampling studies, and examine the extent to which it can reproduce the various manifestations of affect, comparing it with two vector AR models fitted in continuous time. The findings indicate that the AIM model is better at mapping onto manifestations of affect data (especially non-Gaussian manifestations) than the AR models, and it has a higher predictive value for the time series of affect.

I liked this paper very much. I think it can make a very important contribution to our theoretical understanding of affect dynamics.

Thank you for the positive words.

Remarks and concerns:

- My main concern has to do with the format. In its current form, the paper is a bit overwhelming. It is excessively long, with a very large number of figures (19 currently). Some sections read more like a technical report, whereas other sections (i.e., implementation of the model) are more manuscript-type, and very engaging. A full description of the model and its underpinnings needs space, and at times it feels like part of the supplementary material should be included in the main paper as well. On the other hand, combining this explanation and the technical details with the implementation of the model to the two data sets, makes the paper a bit unwieldy. I wonder if it would make sense to split the manuscript into two papers. One could be a more technical paper with all the necessary details, including those from the appendix about fitting the model. The other paper could focus on the implementation of the model to the empirical data sets, comparing the results with those from the two AR models, and fleshing out the implications in terms of affect dynamics.

  The purpose of the paper is to introduce the Affective Ising Model as a model that could be useful and meaningful for analyzing longitudinal data on affect. Although we agree that combining the explanation and the application of the model to the two data sets is a lot of information, we

also believe one of the strengths of the paper to be the fact that it shows that the model can be applied to data and that it can serve as a powerful tool for data analysis. In our opinion, many models are presented that do not consider the goodness-of-fit to actual data.

However, in order to simplify the paper, we have relocated the section on the summary statistics (page 22 and 36) to the appendix. The results on the Gaussian features can be captured both by the AIM and the simpler models, and hence they basically serve as a benchmark for which the fit should be good. In the main text, we now focus on the results regarding the non-Gaussian features for which large differences can be observed between the models. We only summarize the results regarding the Gaussian features (page 22, lines 455-463). In the Materials and Methods section (page 36, lines 836-846) we briefly mention the Gaussian features that have been analyzed and what method we have used. Because of this decision, the number of figures in the main text has been reduced to 12 instead of 19, and the length of the paper has been significantly reduced. We believe this has improved the readability of the paper.

Reviewer #2

In “The Affective Ising Model: A computational account of human affect dynamics”, the authors present a novel computational model for describing and predicting dynamics in self-reported affect. In the process of introducing their model, they analyze two sets of experience sampling data in order to demonstrate the ability of the Affective Ising Model (AIM) to accurately capture observed self-reported affect dynamics and compare the AIM’s performance to other widely used methods of modeling such data. Overall, the manuscript is well-written and makes an interesting and important contribution to the affective sciences.

Thank you for the positive remarks.

However, there are a number of issues that I believe deserve greater consideration and clarification. The authors should provide additional explanation/resources to aid other researchers in the practical implementation of their model.

Additionally, the current manuscript does not adequately address how the model and its parameters should be understood and interpreted in relation to other work or what findings might suggest either future revision or falsification.

Last, there remain questions of the model’s predictive abilities, stemming from the fact that the model is likely most properly understood as a model of dynamics in self-reported affect. I elaborate each of these points below. Overall, I am enthusiastic about the work that the authors have done and believe that efforts to incorporate computational modeling to dynamics in experience sampling such as this will be critical for moving work in this area forward.

Thank you for the critical yet constructive remarks; we provide detailed feedback below.

Remarks and concerns:

- The authors do a noteworthy and commendable job of describing their model in a way that facilitates an intuitive understanding for readers. In fact, it's so intuitively described that it's almost easy to overlook the challenges of practical implementation. To enable other researchers (especially those who are newer to these sorts of models) to use and critique novel computational models, it is important for authors to provide access to materials such as code. This is especially useful because many researchers find actual code as the clearest means for learning the ins and outs of a model, aside from the more formal mathematical description. Therefore, I request that the authors provide access to code for their model. While the full code would likely be the most helpful, a stripped-down version and/or pseudocode, if possible, (e.g., as in Turner et al., 2013, Psychological Methods) could also be helpful. Additionally, a supplement detailing practical challenges, requirements, and limitations for implementing the model would be beneficial. Presenting a model such as the AIM without discussing any of the practical aspects will limit its usage among other research groups.

  Indeed, we agree that it is important to provide other researchers with the necessary means to be able to implement the model. That would be the only way for others to use and fully understand the model, and study its strengths and limitations. This involves sharing code and a user-friendly manual for the software. Currently, we are working on this and soon we will provide both user-friendly software as well as an easy-to-follow manual. However, including it in the current paper (in a way that it can be applied easily) will lead to a much longer paper (and this would go against the recommendations of reviewer 1). Therefore, we have decided not to include any manual or software in the current version of the paper.

- On the topic of clear explication of their model, it was not entirely clear to me how inputs to the model are handled. The authors explicitly state that they avoid discussing inputs in this manuscript, but I believe a discussion of how this might be done warrants greater discussion. Could the authors elaborate on how external events might be fed into the model? Can they provide some proposals for how researchers might code external events? The discussion of this (lines 817-822) could be developed a bit further.

  Thank you for pointing this out. Indeed, the manuscript did not adequately address how input can be handled within the framework of the AIM. When we introduce the basic concepts of the model in the section *Notation* (page 8-9), we mention that the system of binary neurons can respond to external events. What the model would look like when the input strengths $B_1(t)$ and $B_2(t)$ are accounted for was, however, not made explicit. As such, it was difficult to understand exactly how the model handles external inputs. To resolve this issue, we have added a short explanation of what the free energy would look like when the (time-varying) input strengths $B_1(t)$ and $B_2(t)$ are included (page 10, lines 223-227). This will help the reader to understand how input are handled on a mathematical level.

In the *Limitations and future developments* section (page 26-28, lines 575-596), we discuss the difficulty of modeling contextual input in the context of ESM studies. The main issue is that we generally do not have continuous information regarding contextual input at our disposal. Rather, we acquire bits and pieces, and information regarding events are likely to be reported some time after the actual event happened. Particular input functions (e.g., boxcar functions thats assume the stimulus strength to be constant in between measurements) can be used to model such contextual input at the level of implementation. However, one has be aware that this constant strength corresponds to some average strength of all the events that occurred in between measurements – at the time of the measurement, we only detect the accumulated effects of what happened. These are things that have to be considered when trying to model an affect system in interaction with its environment.

Experiments with controlled stimuli (e.g., watching a film fragment or listening to a music piece) might provide a better way of studying and modeling an affect system in interaction with its environment. Stimuli in such experiments can be coded and different participants can be given exactly the same stimuli. People's responses to specific stimuli can be studied and compared to prediction made by a model like the AIM.

- The authors spend a significant portion of the Model Description section justifying the AIM as a computational model that seeks to describe macroscopic phenomena and not the "microscopic" component processes. To help clarify the objectives of their model to readers, it would likely be useful for the authors to draw on already established frameworks such as Marr's (1982) levels of analysis, as has been done elsewhere in the emotions literature (e.g., Adolphs & Andler, 2018; Bach & Dayan, 2017). Using Marr's labels, it seems then that the AIM is meant for an algorithmic level of analysis as it does not appear to make claims about either the purpose of the algorithm or its neural implementation.

  Marr (1982) distinguished between three levels of analysis: the computational level (what is computed and why?); the algorithmic level (how is it computed?); and the implementational level (how is it implemented on a neural level?).

  The Affective Ising Model can be situated at the level of both the algorithm and the implementation. On the implementational level, the model assumes that there exist two separate populations of neurons that respectively process information regarding positive and negative affect. Hence, the model assumes the neuronal network underlying affect to be that of a two pool attractor network. The multiple attractor network is standard paradigm in computational neuroscience.

  On the algorithmic level, the AIM links the affect state of an individual (the affect the individual experiences) to the activities of the pools. It describes the evolution of this affect state as a diffusion process on a free energy surface. The model makes use of the free energy to determine the distribution of attainable affect states given the underlying configuration of underlying attractor network, and describes how the system evolves

from one state to another. Essentially, the affect state tries to minimize the free energy surface, but it fluctuates due to a inherent stochasticity of the network. The shape of the free energy surface can change due to external input, or learning processes that take place over very long time scales (in comparison to the time scale of typical, daily dynamics). When the shape changes the minimum of the surface changes, and therefore the distribution of attainable affect states. This allows the model to describe an intricate interplay between person specific network configurational parameters and external events.

However, the model does not tell us why affective experiences are computed and so no claim is made about the computational level. Obviously, affect is essential for an individual to be able to evaluate its environment and the context, and to determine how to respond. The only goal of the model is to adequately describe the time dynamics of that evaluative signal. In a later stage of the research, when enough support has been collected for the AIM (or a related model), the why-question could be taken up.

Because we agree with the reviewer that this explanation could be interesting for readers who are familiar with Marr's concepts, we have added a paragraph to the section *Bais principles and ideas* (page 7-8, lines 151-160).

- Related to the question of levels of analysis is the question of how the authors believe their model might be informed by research at other levels of analysis. Given the emphasis on the fact that the AIM is intended to capture macroscopic phenomena and not their microscopic components, it would be useful to know whether or not the authors expect research at other levels of analysis to inform, modify, or constrain their model in the future. This question ties into a broader question about falsifiability. For example, are there "affective landscapes" which the AIM cannot produce? (e.g., tri-modal?) As the authors show, constraining the values of the "inhibition" parameter rules out landscapes where PA and NA are positively correlated. However, the participants in their own dataset who showed a positive correlation and other research (e.g., arousal-focused individuals; Barrett & Bliss-Moreau, 2009) would seem to suggest that this parameter constraint should eventually be relaxed.

  This is an interesting point. To derive the dynamics of the AIM, we assumed that the microscopic affect system that resides in the brain and body can be partitioned into two large distinct subsystems, the so-called PA and NA pools. This assumption is supported by both psychological and neurobiological findings (page 5, lines 97-103). It is nonetheless possible that future research uncovers another important dimension that is required in order to capture affective experiences. Maybe the biological structures for processing affect consist of three distinct subsystems. The idea of more than two principal affect dimensions (e.g., an additional arousal dimension) has already been put forward by some researchers. Should it be the case that more pools are required to properly describe affective experiences, then the AIM will have to be extended to more than two pools.

On a different level, there are patterns in the data imaginable that cannot be captured or explained by the AIM. For instance, since the AIM describes a diffusion process on a potential (the free energy surface), it assumes detailed balance. This means, among other things, that affect does not exhibit any cyclic behavior according to the AIM. However, take the example of a crying baby (high NA, low PA). It is possible that the baby always has to go through a neutral state (low NA, low PA) before it can become a happy baby (low NA, high PA). When it is happy, however, it might be able to jump immediately to a sad state and start crying again. Such directional asymmetries in the dynamics violate detailed balance. The only way they are allowed is if they are driven by external inputs.

Regarding the reviewer's remark concerning the inhibition parameter. While rare, there are indeed participants showing a positive correlation between the PA and NA dimensions. If desired, the constraint of inhibition can be relaxed. To see what would happen to our results, we repeated our analyses without this constraint. Due to the rarity of mutual excitation, the results reported in the paper remained largely unaltered (except for the cutoff for the PA-NA correlation).

- The authors do not provide much in the way of discussion of possible psychological interpretations of the model parameters. Perhaps this is because the authors expect that there are no direct concept to parameter mappings, only indirect (e.g., Roberts & Hutcherson, 2019). If this is the case, the authors should be explicit about the reasons for their reticence to speculate about how their model's parameters connect to psychological concepts so that readers can be directed to use the same caution. Do the authors believe that no psychological interpretations should be given to the model parameters and that only features of the resulting "affective landscape" should be given psychological meaning (e.g., the possibility of multiple homebases in patients with borderline personality disorder mentioned in lines 842-847)? The manuscript should offer greater clarity about how the model might be used and interpreted as any readers will certainly want to assign psychological meaning to the parameters.

  It has been correctly observed by the reviewer that we are reluctant to give (psychological or physiological) interpretations to the parameters. The reason is simple: this is the first presentation and empirical test of the AIM. At this stage, we have only proposed a statistical model that mimics empirical affect data, and that is based on solid theoretical and empirical ideas. However, the model has not yet sufficiently firmly been established in order to give definite meaningful interpretations to the parameters. Future empirical research can help further establish the model. For example, experiments could be set up that target a specific parameter (only this parameter and not the others). This is called selective influence. Such a test for selective influence could gives us more confidence about the interpretation of the parameters. Another possibility is that we discover links between the parameters and certain physiological characteristics.

  Given that selective influence has not yet been established and that no specific links to physiology have been made, we should be careful with

interpreting the parameters. We could easily apply a parameter transformation, leading to a different set of parameters (with totally different interpretations) but producing the same probability density functions and dynamics, and thus fitting the data equally well.

Even though it is not (yet) possible to assign psychological or physiological interpretations to the model parameters, the strength of the AIM is that it encompasses numerous non-trivial distributional features. As far as we know, there exists no affect model that is able to unify the non-Gaussian features described in the paper (skewness, V-shape and bimodality) in a single dynamical framework. These features have indeed been hypothesized to be important for the characterization of specific psychopathologies related to affect. As mentioned by the reviewer, patients with borderline personality disorder are believed to typically have multiple homebases. In general, it is difficult to validate such hypothesis because a lot of data is required to reveal non-Gaussian features. There exist statistical tests for detecting bimodalities in data, but they often rely on specific assumptions and none of them are waterproof. In addition, they are often limited to univariate distributions. Provided the AIM is a good model for affect, it can be used to uncover such non-Gaussian features and help validate specific claims that have been made about the occurrence of them in specific clinical groups.

A paragraph regarding the comparison of individuals using the model has been added to the section *Limitations and further developments* (page 26-28, lines 609-641).

- The authors compare their model to two other models that are Gaussian, with or without bounds. One of the highlighted strengths of the AIM is its ability to deal with the boundedness of the data and produce the observed skewness. However, this demonstration raises the unanswered question of to what degree these aspects of the data are due to affective dynamics versus due to how people use the sorts of self-report scales they are provided. To be clear, I do not doubt that affective experience is non-normally distributed. However, it seems highly likely that some of the distributional features of the data are also contributed to by the self-report measures themselves. For example, though participants may use the most extreme response for negative affect on an occasion, is this capturing their most extreme possible level of negative affect? Similarly, though participants appear to frequently report no negative or positive affect at all, is this truly their "flattest" affective experience? All of this suggests the subtle, yet important, distinction between a model of affective dynamics versus a model of dynamics in self-reported affect. I do not see this point as an invalidation of the AIM, but realizing this distinction raises important additional questions. For example, if the AIM is a model of dynamics in self-reported affect, how might a person's "affective landscape" be influenced by changes to the self-report measure (e.g., scale anchors, items, etc.)? Will fitting the AIM to an individual's data collected using one self-report measure allow for accurate predictions of data collected using a different measure? If there are discrepancies in how the model fits to data derived from different measures, how should these be dealt with? It

would seem important for the authors to acknowledge these questions and how they might be addressed.

These are worthwhile points made by the reviewer. The data analysed in the paper have been collected under a specific protocol, involving decisions regarding the emotion items that were included in the questionnaires, the response format (continuous sliders), labels that were used, the specific formulation of questions and the sampling rate. The features described in this paper are likely to be influenced by these choices. In this paper, we did not wish to make any substantial claims about the features themselves. We merely pointed out that features like skewness, V-shapes and bimodalities have been repeatedly reported in data (they were also common in the data we analysed).

The goal of the paper is mainly to introduce a model that is able to unify such non-Gaussian features in a single dynamical framework and to show that the AIM is a viable model for affect dynamics. To achieve this goal, we had to connect affect data to the model description of affect in terms of average activities of pools. Our approach (to average positive and negative affect scores) was based on techniques that are common in the research field, but it not necessarily the best approach. Our approach with regards to measurement noise and contextual input is probably also too simplistic to make accurate substantial claims about affect dynamics.

Because of the importance of these issues, we address them in the section *Limitations and future developments* (page 26-28, lines 550-574). We also mention that some of these issues can be resolved by complementing the AIM with a proper measurement model, using item response theory for example, that incorporates measurement noise.

Minor Remarks:

- On lines 103-106, the authors state "Most likely the biological structures dealing with positive and negative affect information consist of a large number of smaller elements (neurons, neurotransmitters, hormones, etc.) that in interaction with psychological processes produce positive and negative feelings." I acknowledge that my objection here is possibly a bit idiosyncratic, but I always have trouble understanding what is meant by a sentence such as this. Presumably the authors agree that biological structures such as neurons, neurotransmitters, and hormones are part of the material substrates for psychological processes. So what is meant by neurons interacting with psychological processes? To me this sounds like a subset of elements interacting with their superset, which does not make sense to me. Do the authors mean to say something about how psychological processes are constrained by aspects of their biological underpinnings? Or how features of the biological structures interact with an organism's broader ecology and history?

  After re-reading the sentence, we agree that it may come across as vague and confusing. The main message that we want to convey is that we believe that biological structures such as neurons, neurotransmitters and hormones are part of the material substrates for psychological processes

and that a psychological process (like processing affect information) is the result of the interactions of an incredibly large number of such biological structures. What was meant with the part ‘in interaction with psychological processes’ is that other psychological processes (cognitive processes for example) can influence affect information processing. Of course, like the reviewer correctly points out, these other psychological processes also have their origin in material substrates such as neurons, neurotransmitters and hormones. To avoid such confusion, we have decided to leave out the part ‘in interaction with psychological processes’ resulting in the sentence: “Most likely the biological structures dealing with positive and negative affect information consist of a large number of smaller elements (neurons, neurotransmitters, hormones, etc.) that collectively produce positive and negative feelings.” (lines 103-106)

- The sentence on lines 189-191 appears to be missing a “the”: “As a result, absorbing such an input process into THE model does not affect the model’s properties (e.g., structure, dynamics, distributions, etc.).”

  We are grateful to the reviewer for detecting this error. The ‘the’ was added to the sentence (line 200).
